# Effects of Patient Portal Use on Patient Satisfaction: Survey and Partial Least Squares Analysis

**DOI:** 10.2196/19820

**Published:** 2021-08-27

**Authors:** Aaron P Kinney, Balaji Sankaranarayanan

**Affiliations:** 1 Herma Heart Institute, Children's Wisconsin Milwaukee, WI United States; 2 Department of Information Technology and Supply Chain Management, College of Business and Economics, University of Wisconsin - Whitewater Whitewater, WI United States

**Keywords:** patient portal, patient satisfaction, gratification, health self-awareness, post-adoptive use, health perceptions

## Abstract

**Background:**

With digital delivery of health care services gaining prominence, patient portals have become a mainstay of many health care organizations. Despite the importance of patient portals, inconclusive data exist regarding the effect of patient portal use on patient satisfaction.

**Objective:**

The aim of this study is to understand the relationship between the postadoptive use of patient portals and patient satisfaction outcomes.

**Methods:**

Postadoptive use of patient portals has a positive relationship with the 3 dimensions of patient satisfaction, mediated by gratification, health self-awareness, and health perceptions. A total of 504 valid patient portal user responses were collected, and partial least squares analysis was performed to analyze the data.

**Results:**

Patient satisfaction was captured using three dimensions: care team interaction, atmosphere, and instruction effectiveness. The results show that postadoptive use of patient portals has a positive influence on all 3 dimensions of patient satisfaction through the mediating variables of gratification, health self-awareness, and health perceptions. Specifically, postadoptive use had significant positive influence on gratification, health self-awareness, and health perceptions. Each of the 3 patient perceptions had significant positive influence on all 3 dimensions of patient satisfaction: care team interaction, atmosphere, and instruction effectiveness. Specifically, our model explained 31.8% of the care team interaction, 40.6% of the atmosphere, and 39.1% of the instruction effectiveness.

**Conclusions:**

Our model shows that patient portal use can influence patient satisfaction through the mediating effects of gratification, health self-awareness, and health perception. Patient satisfaction is an important outcome for health care organizations. Therefore, by promoting effective patient portal use and fostering patient perceptions, health care organizations can improve patient satisfaction.

## Introduction

### Background

Increasingly, as health care digital services have gained prominence, patient portals have become a mainstay of many health care organizations. The impetus for implementing patient portals can be traced to the Affordable Care Act and Health Information Technology for Economic and Clinical Health Act, which tied reimbursements to the implementation of health information infrastructure and achievement of benchmark patient satisfaction scores [1]. Practitioner reports state that 90% of the health care organizations had implemented a patient portal in some form by 2018 [2]. The portals’ services range from providing offline health records, messaging, and alerts to providing web-based real-time doctor consultations [3]. However, this leads to the key research question: does a patient’s use of a portal lead to increased patient satisfaction?

As a performance metric, patient satisfaction scores are extremely important for health care organizations. Patient satisfaction scores, also known as Hospital Consumer Assessment of Healthcare Providers and Systems (HCAHPS) scores [4], affect the payments made to health care organizations [1]. In addition, clinical benefits such as improved clinical outcomes and improvement in overall clinical care constitute a significant benefit of higher patient satisfaction [4]. Although satisfied patients gain these significant benefits, dissatisfied patients can ignore—or worse, completely abandon—the care provided. As patient portals continue to gain prominence, improved patient satisfaction can enable organizations to provide effective health services through patient portals. Thus, studying the link between patient portal use and patient satisfaction becomes an extremely interesting and important research problem [5].

Despite strong academic interest in patient satisfaction as a performance metric, up to 45% of the current studies on the effect of portals on patient satisfaction have either shown no effect or have been inconclusive [6]. Although patient portals have been found to be associated with better patient retention [7], increased patient care compliance [8], reduction of medication errors [9], and improvement in communication [10], these relationships do not seem to have a direct link to the HCAHPS dimensions of patient satisfaction. Although prior findings on the benefits of patient portals are unequivocal, the relationship between portal use and patient satisfaction is still unclear.

Our study seeks to address this research gap in 2 ways. We theorized and tested a research model linking patient portal use and the *HCAHPS dimensions* of patient satisfaction. Furthermore, to address the research gap in our understanding of the link between portal use and satisfaction, we theorized on the mediators that link portal use and patient satisfaction. Using the Adaptive Structuration Theory (AST) and prior studies, we modeled patient perceptions as mediators of the relationship between patient portal use and patient satisfaction. In the following section, we elaborate on the prior studies on patient portals. Next, we describe how we built and tested a research model of patient portal use, patient perceptions, and patient satisfaction.

### Prior Work

Our literature review revealed several important themes regarding patient portal use and its impacts. Some key articles on patient portals and their impacts are presented in Multimedia Appendix 1 [1,3,6,7,9-20]. The factors that predict the adoption and use of patient portals have received extensive attention in the literature. The Technology Acceptance Model (TAM) and the Unified Theory of Acceptance and Use of Technology (UTAUT) offer interesting theoretical perspectives to study the implementation of patient portals. Research applying this framework shows that perceived value and ease of use are important predictors of portal adoption [5-8]. The rationale is that patient portals offer the ability to access data, scheduling, and reports at any time (perceived usefulness), in addition to allowing certain functionalities without any of the previous barriers (ease of use), such as scheduling an appointment without waiting on a callback. Recent research also acknowledges that adoption is different from use and that adoption may not necessarily translate into the use of a patient portal [9].

Although the UTAUT and TAM provide an excellent framework to examine the phenomena of *adoption*, there is a significant gap in this research regarding the impacts of the system *after* use, that is, postadoptive use. Several studies have attempted to address this research gap by focusing on the relationship between patient portal use and patient outcomes. The portal benefits of improving care and communication between patients and providers [10,11], discovering medical errors, and ensuring that patients take medication on time [12] have been documented in the literature. However, findings from review studies also confirm the inconsistency in patient satisfaction studies [6]. Although some studies have found improved patient satisfaction when electronic patient access to medical information was used [6,13,14,21], other studies have found mixed results [7,15,22], and a few have found that patient satisfaction data remained unchanged [23,24].

Two important aspects of prior research on patient satisfaction must be mentioned. First, although using HCAHPS measures are considered the standard way to measure patient satisfaction at health care organizations, patient satisfaction has not been measured consistently, or it has been measured using only 1 or 2 HCAHPS measures [14,16]. This implies that patient satisfaction needs to be studied more comprehensively by enumerating its underlying dimensions. This will provide a richer description of the influence of patient portals.

Second, because of the research gap in the literature regarding the link between patient portal use and patient satisfaction, the mediators linking these factors need to be considered. Prior research suggests that patient perceptions could play a mediating role in determining patient satisfaction through patient portal use [7,14]. Patient portal use can influence a positive patient experience for patients [3]. Patients with chronic disease have mixed attitudes regarding patient portal use [17]. This suggests that studying the mediating role of patient perceptions could help address the link between patient portal use and patient satisfaction.

### Goal of This Study

Prior studies show that patient satisfaction and patient portals have many important intertwined relationships to be explored. Although patient portals have been explored in terms of their impact on different aspects of the care continuum, there remains an inconsistent understanding of the relationship between portal use and patient satisfaction. As explained earlier, an understanding of the mediating influences of patient perceptions will shed light on the links between patient portals and patient satisfaction. Therefore, the 2 research questions addressed in this study are as follows:

1. What are the influences of patient portal use on patient satisfaction?
2. What are the influences of patient portal use on patients’ perceptions of their own health and portal?

### Research Model and Hypotheses Development

The AST [25] serves as the theoretical perspective for our research model. The AST has been applied to study adoption perceptions and behaviors in a variety of contexts such as group decision support systems and enterprise systems. Drawing upon the structuration theory formulated by Giddens [26], the AST offers a theoretical framework that delves into the dynamic relationship that connects *structures* provided by technology and the ways in which these structures are *appropriated or adapted* by a user. Traditionally, the AST had been applied in the organizational or group context [25]. However, recent research has adapted this framework to the individual level [27]. Applying the AST to the individual level, the input-process-output framework of the AST can be explained as follows: (1) input: technology in use affords certain features or structures; (2) process: technology is adapted by users to accomplish a task, and users develop certain attitudes, perceptions, and behaviors; and (3) output: processes can influence decision-making or performance outcomes [27].

**Figure 1:**
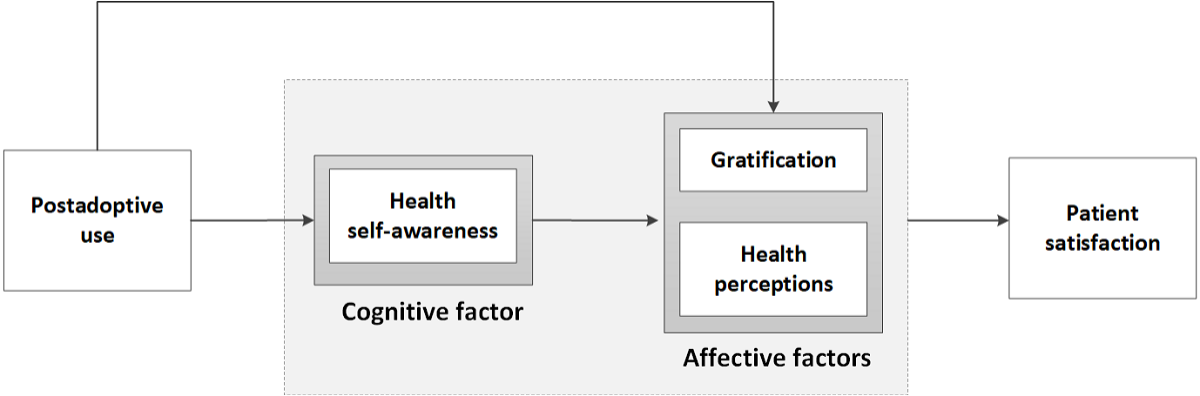
Influences of postadoptive use of electronic portals on patient perceptions and satisfaction.

Adapting the AST’s input-process-output framework in our study, we posited that (1) patient portals afford certain technology features for use, (2) portal use can influence patient perceptions, and (3) patient perceptions can influence patient satisfaction outcomes. The research model of patient portal use and patient satisfaction is presented in Figure 1. The model shows that postadoptive use will have a positive influence on health self-awareness, gratification, and health perceptions. We classified them as cognitive factor (knowledge-based) and affective factor (emotion-based). These patient perceptions are posited to have a positive influence on patient satisfaction.

### Dimensions of Patient Satisfaction

As noted earlier, patient satisfaction has not been conceptualized or measured consistently as an outcome variable in prior research. It is common for numerous measures of patient satisfaction to be used, including likeliness to recommend [28], satisfaction with nursing [29], and satisfaction with physician communication [30]. Some studies have only used 1 or 2 items from the HCAHPS survey (either overall satisfaction or willingness to recommend) to measure patient satisfaction [14,16].

We diverged from these studies to conceptualize patient satisfaction as a multidimensional construct using multiple measures. The conceptualization and measures used in this study for patient satisfaction were based on the HCAHPS survey. This is the national survey used by health care organizations to capture patient perceptions of hospital experience and is used by the Centers for Medicare & Medicare Services to standardize medical reimbursement [4]. This survey is typically used to gauge patient satisfaction at hospitals [31] and has been a critical component in bringing about transparency to patient perceptions of care [32]. On the basis of the HCAHPS survey, patient satisfaction was theorized in this study as consisting of three dimensions: care team interaction, atmosphere, and instruction effectiveness. Care team interaction refers to any communication between a patient and a member of the care team, such as providers and nurses. Atmosphere refers to the evaluation of the health encounter with respect to the environment around the patient, notably cleanliness, quietness, and staff responsiveness. Instruction effectiveness captures the care team’s ability to convey pertinent information to the patient, including communication about medicines and discharge information.

### Health Self-awareness

In this study, health self-awareness was defined as the extent of knowledge and skill sets of patients in relation to their own health. Prior research has shown that patients are more informed about their health through patient portal use [11,33]. As patients continue to use patient portals, these technology features [25] provide them with access to accurate information about their health status [34]. In addition, patient portals provide detailed explanations of the test results and associated health conditions [19]. Patients can therefore compare such information (eg, blood work results) against established benchmarks, in addition to obtaining a detailed understanding of their health condition. As patients accumulate knowledge about their health, they are more likely to be informed and involved in their health care decisions [35]. Informed patients can proactively request specific health services from their providers [36], which can reduce medical errors [12,34], improve decision-making [37], and subsequently influence the 3 dimensions of patient satisfaction (care team interaction, atmosphere, and instruction effectiveness). Hence, we postulated the following hypotheses:

- H_1_: Health self-awareness mediates the positive influence of postadoptive use on patient satisfaction.
- H_1a_: Postadoptive use will have a positive influence on health self-awareness.
- H_1b_: Health self-awareness will have a positive influence on patient satisfaction.

### Gratification

Gratification has been defined in numerous ways, particularly in relation to the Uses and Gratification Theory [38,39]. In this study, we defined gratification as a feeling of pleasure directly related to achieving a desired task such as scheduling, reviewing medical information, or using the patient portal. Patient portal use provides a near-instant ability to achieve health-related tasks [40,41], rather than limiting individuals to accomplishing such tasks only during business hours [42]. As the goal of portal use is information seeking or knowledge gathering [41], the immediate sharing of information is likely to gratify the patient. Gratified patients will reflect positively upon their care experience [41,43], which will have a positive influence on their satisfaction with overall health care delivery. Hence, we posited the following:

- H_2_: Gratification mediates the positive influence of postadoptive use on patient satisfaction.
- H_2a_: Postadoptive use will have a positive influence on gratification.
- H_2b_: Gratification will have a positive influence on patient satisfaction.

### Health Perceptions

In this study, health perceptions were defined as having positive emotions about a person’s health. Prior work in psychology literature has shown the importance of positive emotions as they improve creative problem solving [44] and satisfaction [45] and increase the likelihood of success [46]. Research in information systems literature has also pointed to the centrality of positive emotions in predicting the use of systems. For instance, knowledge gained through the UTAUT [47] pointed to affect and associated constructs such as computer playfulness as antecedents of behavioral intention to use a system.

We posited that health perceptions would mediate the relationship between postadoptive use and patient satisfaction. First, prior studies have informed us that health perceptions can influence outcome variables, including practice satisfaction in physicians [45,46,48]. As users gather information, they experience positive health perceptions, and this affective attitude component directly contributes to their satisfaction attitudes. As long as the information technology system (ie, portal) continues to enable this behavior [25], it has the potential to allow users to feel more positive about their health. As patients have more positive feelings about their health status, they are more likely to rate the status of their health experience higher, leading to higher patient satisfaction scores. Hence, we posited the following:

- H_3_: Health perceptions mediate the positive influence of postadoptive use on patient satisfaction.
- H_3a_: Postadoptive use will have a positive influence on health perceptions.
- H_3b_: Health perceptions will have a positive influence on patient satisfaction.

### Indirect Effects of Health Self-awareness

Health self-awareness can have a positive impact on gratification and health perceptions, thereby indirectly influencing patient satisfaction. On the basis of social cognitive theory [49], prior research in different contexts has shown that cognitive factors can influence affective factors. For example, in a learning context, working memory skills (cognitive factor) can influence writing anxiety and self-efficacy (affective factors), further affecting writing performance [50]. Similarly, in a health care context, mindfulness, a cognitive factor, can have a positive impact on affective empathy, leading to improved engagement in nursing [51]. As explained earlier, as a patient’s knowledge increases, they are more likely to be informed and involved in their own health care decisions [35]. As patients begin to make informed decisions, it can lead to a higher level of gratification (ie, satisfaction with learning through technology use) and health perceptions (ie, a positive feeling about taking control of their own health). On the other hand, as health self-awareness decreases, the patients’ capacity to make informed decisions also decreases, which can lead to lower levels of gratification and health perceptions. Hence, we posited the following:

- H_4_: Health self-awareness will have an indirect influence on patient satisfaction through gratification and health perceptions.
- H_4a_: Health self-awareness will have a positive influence on gratification.
- H_4b_: Health self-awareness will have a positive influence on health perceptions.

## Methods

### Recruitment

As part of a larger research study, this survey was designed as a nationwide electronic survey to be disseminated within the United States. This study used a Qualtrics panel (Qualtrics) with a financial incentive provided to the respondents (the negotiated rate with Qualtrics was a little less than US $5 per respondent). Qualtrics is a highly reputed experience management company that provides a platform for survey design and execution. It maintains a panel of respondents and recruits them depending on the purpose of the survey. For our study, we sought respondents who had visited their regular health care facility and used an electronic patient portal within the last 12 months. Respondents were excluded from participating in the survey if they did not meet these 2 criteria. Regular health care facilities were defined in the survey as health care facilities (eg, primary care provider’s office and hospital) that the respondents typically visit for health care services. The survey was expected to take approximately 15 minutes to complete.

Electronic patient portals were defined as the secure websites of the regular health care facilities that provide patients with convenient 24-hour access to their personal health information such as recent or upcoming medical visits, prescriptions, and vaccinations. Furthermore, the instructions also stated that participation in this research was anonymous and voluntary. The exclusion criteria also included respondents failing to consent to take the survey, failing to confirm that they were aged above 18 years, or failing to complete the survey. A final sample of 504 responses was obtained.

### Item Development and Expert Review

Item development for this study began by using established measures. When the established measures could not be used or were modified to the extent that the expert review suggested that they be treated as new items, new items were established in a rigorous process (Textbox 1). Prior research has laid clear blueprints for item development for this survey [52,53]. For each new item, initial development was informed by a literature review and the close collaboration with an experienced information technology professor with experience in health care research. A panel of subject matter experts related to the topic at hand was then created, including physicians, nurses, health care administrators, and health care researchers. After gaining insight from the expert review and making adjustments, a pilot study was completed, consisting of 20 health care staff members, 9 of whom completed the survey. These results were used to modify and finalize the survey instruments. Subsequently, an additional pilot survey was conducted using 43 surveys completed by doctoral students.

**Measures and sources.**

**Patient satisfaction (17 Items)**

- Adapted from [4]

**Postadoptive portal use (4 Items)**

- Adapted from [54]

**Health self-awareness (3 Items)**

- Adapted from [54]

**Gratification (3 Items)**

- Developed new items based on [38]

**Health perceptions (4 Items)**

- Adapted from [54]

### Common Method Bias

As this study measured predictor and criterion variables using the same system, time, and source [55,56], it was necessary to test for common method bias as it increases the possibility of inflated results [55,56]. Statistical tests for common method bias [56] suggested that it may not be a significant concern in this study.

### Control Variables

The control variables measured in this survey included age, gender, income, education, and race. We also modeled health anxiety as a control variable. Health anxiety was captured by the item *I am very anxious about my health* on a strongly disagree to strongly agree scale. Race was modeled as a 0-1 variable, with 1 representing Caucasian. Table 1 shows the demographic characteristics of the respondents.

**Table 1:** Demographic characteristics of the respondents (N=504)^a^.

Characteristics		Respondents, n (%)
**Age (years)**		
	18-25	61 (12.1)
	26-30	68 (13.5)
	31-35	60 (11.9)
	36-40	61 (12.1)
	41-45	50 (9.9)
	46-50	37 (7.3)
	51-55	36 (7.1)
	56-60	32 (6.3)
	61-65	31 (6.2)
	>65	66 (13.1)
**Gender**		
	Male	159 (31.5)
	Female	343 (68.1)
**Education**		
	Eighth grade or less	15 (2.9)
	Some high school but did not graduate	115 (22.8)
	High school graduate or General Educational Development certificate	182 (36.1)
	Some college or 2-year degree	122 (24.2)
	4-year college graduate	70 (13.9)
**Income (US $)**		
	≤25,000	107 (21.2)
	25,001-50,000	150 (29.8)
	50,001-75,000	116 (23)
	75,001-10,000	57 (11.3)
	100,001-125,000	33 (6.5)
	125,001-150,000	14 (2.8)
	150,001-200,000	12 (2.4)
	200,001-250,000	2 (0.4)
	>250,000	6 (1.2)
**Race**		
	Caucasian	390 (77.4)
	African American	53 (10.5)
	Asian	12 (2.4)
	American Indian or Alaska Native	8 (1.6)
	Native Hawaiian or Pacific Islander	0 (0)
	Mixed race	7 (1.4)
	Other	26 (5.2)

^a^For each of the demographic variables, missing data constituted the remaining percentage.

## Results

### Overview

We used SmartPLS (version 3.3.2, SmartPLS GmbH) structural equation modeling to analyze the data. The measurement model was first examined to evaluate reliability, convergent validity, and discriminant validity. The structural model was then evaluated to test the specific hypotheses. The measurement model and structural model analysis are presented below.

### Measurement Model

Confirmatory factor analysis was performed to establish the reliability and validity of the measures [57]. The measures and loadings are presented in Table 2. Composite reliabilities were above the threshold of 0.7, and all item loadings were statistically significant and above the acceptable threshold of 0.70 [58]. Table 3 shows the validation of the measurement model for the constructs in this study.

The diagonal elements (Table 3) show the square root of the average variance extracted (AVE). The second-order constructs were modeled in partial least squares following the procedure described in the study by Pavlou and El-Sawy [59]. The dimensions of care team interaction, instruction effectiveness, and atmosphere (Table 2) were modeled as first-order constructs. The model then calculates the path weights from the first-order constructs to the second-order constructs, and latent factor scores are calculated for each second-order construct. As each second-order construct is represented by a latent factor score and not by multiple items, the AVE for all second-order constructs is 1. The off-diagonal elements show correlations among the constructs. The AVE values were found to be greater than 0.5 [60]. This establishes the convergent validity of the constructs. The item-to-construct correlations for each construct were found to be less than the square root of the corresponding AVE, thus establishing discriminant validity [60].

**Table 2:** Constructs, measures, and loadings.

Item				Indicator		Loading		Composite reliability	
**GR^a,b^**									0.951
	GR_1_			I feel satisfied when I receive information regarding my health via the electronic patient portal		0.920			
	GR_2_			Accomplishing health care related tasks via the electronic patient portal when it is convenient for me is satisfying (eg, scheduling appointments, checking test results, etc)		0.930			
	GR_3_			I feel satisfied about my experiences completing health care related tasks via the electronic patient portal (eg, scheduling appointments, checking test results, etc)		0.941			
**HSA^b,c^**									0.947
	HSA_1_			I have a good understanding of my health status		0.947			
	HSA_2_			I am informed regarding ideal targets for indicators of my health (eg, weight, cholesterol, blood sugar, etc)		0.917			
	HSA_3_			I am knowledgeable about my health status		0.942			
**PAU^b,d^**									0.915
	PAU_1_			Please select your usage frequency - scheduling appointments		0.865			
	PAU_2_			Please select your usage frequency - emailing my provider		0.884			
	PAU_3_			Please select your usage frequency - checking test results		0.845			
	PAU_4_			The electronic patient portal is used frequently by me		0.818			
**HP^b,e^**									0.924
	HP_1_			I lead an active and healthy life		0.846			
	HP_2_			I feel optimistic about my health		0.876			
	HP_3_			I feel satisfied with my latest health check-up results (eg, blood pressure, cholesterol, glucose levels)		0.854			
	HP_4_			In general, I am enthusiastic about my health		0.891			
**Care team interaction^f^**									
	**DC^g^**							0.944	
		DC_1_	How often did providers (eg, doctors) treat you with courtesy and respect?		0.918				
		DC_2_	How often did providers (eg, doctors) listen carefully to you?		0.919				
		DC_3_	How often did providers (eg, doctors) explain things in a way you could understand?		0.927				
	**NC^h^**							0.936	
		NC_1_	How often did nurses treat you with courtesy and respect?		0.907				
		NC_2_	How often did nurses listen carefully to you?		0.931				
		NC_3_	How often did nurses explain things in a way you could understand?		0.897				
**Atmosphere^f^**									
	**CQ^i^**							0.916	
		CQ_1_	How often were public restrooms found clean?		0.919				
		CQ_2_	How often was the noise level quiet during appointments?		0.919				
	**SR^j^**							0.866	
		SR_1_	How often did you get help from any staff as soon as you wanted it?		0.818				
		SR_2_	How often did you get an appointment as soon as you needed?		0.858				
		SR_3_	When contacting my typical health care facility with a question, I typically received an answer the same day		0.802				
**Instruction effectiveness^f^**									
	**CM^k^**							0.844	
		CM_1_	How often did hospital staff describe possible side effects?		0.810				
		CM_2_	How often did hospital staff tell you what the medicine was for?		0.864				
		CM_3_	I clearly understood the purpose for taking each of my medications		0.730				
	**DI^l^**							0.910	
		DI_1_	Staff took my preferences into account in deciding what my health care needs would be		0.904				
		DI_2_	Whenever I left my typical health care facility, I had a good understanding of the things I was responsible for in managing my health		0.888				
		DI_3_	I received information in writing summarizing the visits and describing any symptoms or health problems to look out for		0.840				

^a^GR: gratification.

^b^Scale used for use frequency questions: 1=never to 7=multiple times a day; scale used for all other questions: 1=strongly disagree to 7=strongly agree.

^c^HSA: health self-awareness.

^d^PAU: postadoptive use.

^e^HP: health perceptions.

^f^Patient satisfaction dimensions: second-order constructs: scale used for “how often” questions: 1=never to 7=always; scale used for other questions: 1=strongly disagree to 7=strongly agree.

^g^DC: doctor communication.

^h^NC: nurse communication.

^i^CQ: cleanliness and quietness.

^j^SR: staff responsiveness.

^k^CM: communication about medicines.

^l^DI: discharge information.

**Table 3:** Measurement model validation^a^.

Constructs	Value, mean (SD)	ATMOS^b^	CTI^c^	IE^d^	GR^e^	HSA^f^	HP^g^	PAU^h^
ATMOS	5.82 (0.98)	*1^i^*	—^j^	—	—	—	—	—
CTI	6.07 (0.99)	0.776	*1*	—	—	—	—	—
IE	5.78 (1.05)	0.787	0.696	*1*	—	—	—	—
GR	5.32 (1.41)	0.453	0.384	0.475	*0.930*	—	—	—
HSA	5.89 (1.16)	0.540	0.496	0.528	0.515	*0.925*	—	—
HP	5.19 (1.31)	0.398	0.321	0.444	0.386	0.520	*0.867*	—
PAU	3.54 (1.47)	0.076	−0.012	0.148	0.458	0.155	0.237	*0.853*

^a^Latent scores of second-order constructs (atmosphere [ATMOS], care team interaction [CTI], and instruction effectiveness [IE]) are standardized scores. Hence mean and SD are 0 and 1, respectively. However, in this table, we have provided the mean and SD of all the corresponding items for ATMOS, CTI, and IE.

^b^ATMOS: atmosphere.

^c^CTI: care team interaction.

^d^IE: instruction effectiveness.

^e^GR: gratification.

^f^HSA: health self-awareness.

^g^HP: health perceptions.

^h^PAU: postadoptive use.

^i^The diagonals show the square root of the average variance extracted. Diagonal values for second-order constructs (atmosphere, care team interaction, and instruction effectiveness) are 1 because these are modeled using latent factor scores. Off-diagonal elements show correlation among the constructs.

^j^Not applicable.

### Structural Model

**Figure 2:**
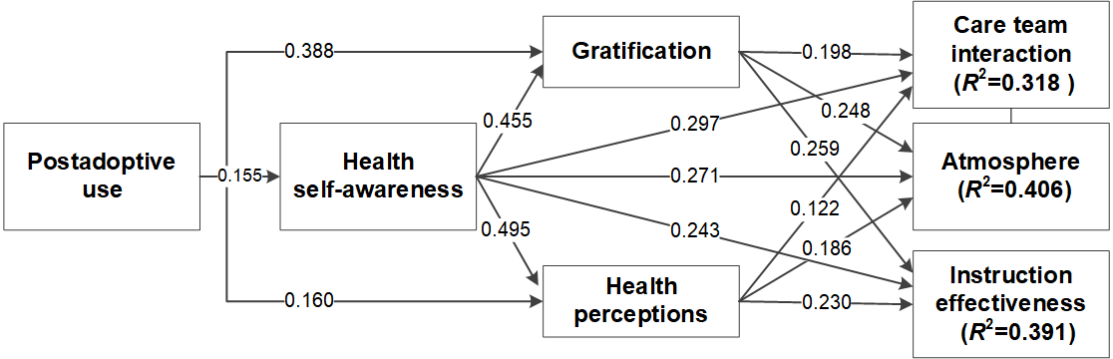
Structural model of influences of postadoptive use.

Figure 2 shows the structural model with all the *β* values and significance of the paths. Care team interaction, instruction effectiveness, and atmosphere were modeled and validated as second-order factors [59]. The items associated with the second-order construct were first checked for convergent and discriminant validity. Next, the path coefficients from the first-order constructs to the second-order constructs were checked for significance. The corresponding path coefficients were found to be significant at *P*<.001: doctor communication to care team interaction (*β*=.537); nurse communication to care team interaction (*β*=.523); cleanliness and quietness to atmosphere (*β*=.488); staff responsiveness to atmosphere (*β*=.633); discharge instructions to instruction effectiveness (*β*=.597); and communication about medicines to instruction effectiveness (*β*=.482). This established the second-order constructs, and the latest factor scores were obtained for each dimension of patient satisfaction. The model was then tested with all constructs and latent scores of the second-order constructs.

Table 4 shows the *β* values and *P* values for each hypothesis. All hypotheses in the research model were supported. Furthermore, the Sobel test for each mediating relationship showed that all mediating influences were statistically significant (Multimedia Appendix 2 [61]). Among the control variables, numerous significant relationships were observed. Age had a significant relationship with atmosphere (*β*=.209; *P*<.001), care team interaction (*β*=.201; *P*<.001), and instruction effectiveness (*β*=.110; *P*=.006). Gender was significantly related to atmosphere (*β*=.091; *P*=.01), care team interaction (*β*=.088; *P*=.03), and instruction effectiveness (*β*=.088; *P*=.02). Race was significantly related to atmosphere (*β*=.088; *P*=.02) and instruction effectiveness (*β*=.091; *P*=.004). Education, income, and health anxiety were not significantly related to any of the dimensions.

**Table 4:** Results of hypothesis testing.

Hypothesis			*β*		*P* value
**H_1a_**					
	Postadoptive use to health self-awareness	.155		<.001	
**H_1b_**					
	Health self-awareness to care team interaction	.297		<.001	
	Health self-awareness to atmosphere	.271		<.001	
	Health self-awareness to instruction effectiveness	.243		<.001	
**H_2a_**					
	Postadoptive use to gratification	.388		<.001	
**H_2b_**					
	Gratification to care team interaction	.198		<.001	
	Gratification to atmosphere	.248		<.001	
	Gratification to instruction effectiveness	.259		<.001	
**H_3a_**					
	Postadoptive use to health perceptions	.160		<.001	
**H_3b_**					
	Health perceptions to care team interaction	.122		.009	
	Health perceptions to atmosphere	.186		<.001	
	Health perceptions to instruction effectiveness	.230		<.001	
**H_4a_**					
	Health self-awareness to gratification	.455		<.001	
**H_4b_**					
	Health self-awareness to health perceptions	.495		<.001	

## Discussion

### Principal Findings

Our findings show that patient portal use has a positive influence on the three mediators: health self-awareness, gratification, and health perceptions. Each of the 3 mediators also has positive influences on the 3 dimensions of patient satisfaction: care team interaction, atmosphere, and instruction effectiveness. This study contributes to our understanding of the influence of patient portal use on patient satisfaction in 3 distinct ways. First, this study addressed an important research question regarding the link between patient portal use and patient satisfaction [25]. This study diverged from earlier studies that have used *few select items* to measure patient satisfaction by using *multiple dimensions* of the HCAHPS measures for patient satisfaction (Multimedia Appendix 3 [14,16,28,29]). This is a novel contribution to extant research because it helps us to discern the portal use influences on the various facets of patient satisfaction. Future studies can use this conceptualization of patient satisfaction not just for patient portal use, but also for other applications.

Second, this study extends prior work on the use of the TAM and UTAUT on patient portals and postadoptive use by highlighting the *pathways* through which portal use can influence patient satisfaction. We have enumerated the role played by three mediators: health self-awareness, gratification, and health perceptions. Through these mediators, this study makes novel contributions to the extant literature and practice in terms of *how* postadoptive use influences patient satisfaction. Notably, our study confirmed the role of health self-awareness as a critical mediator. This study used the AST as the underlying theory because it deals with technology adoption and use behaviors. However, psychological theories such as the Mere Exposure Effect [62-64] could also serve as a broader theoretical underpinning for our study. The Mere Exposure Effect as espoused in the study by Zajanc [62-64] states that a person’s familiarity with, for example, an object would make them develop a preference for it. Applied to the context of the patient portal, a patient’s familiarity with the patient portal can influence them to develop affective reactions, which can lead to patient satisfaction. In other words, efforts to design technologies that facilitate learning and knowledge acquisition can play a critical role in higher patient satisfaction scores.

Furthermore, we defined gratification as a feeling of pleasure directly related to achieving a desired task through technology use, as informed by the Uses and Gratification Theory [38]. In an era when research has demonstrated that consumers seek computer-mediated interactions and that such use can provide gratification [40], it is not surprising to find gratification playing such a critical mediating role. Our study found that as the feeling of gratification is achieved, this affective component directly affects the patient’s satisfaction with the entire care experience, thereby expanding the Uses and Gratification Theory into the patient portal domain. As the patient portal is used extensively, ways to influence this variable seem critically important with regard to influencing patient satisfaction.

Finally, health perceptions were found to play a key mediating role between postadoptive use and patient satisfaction. Prior studies show that health perceptions have an impact on the satisfaction variables [45,46,48]. By empirically validating the influence of patient portal use on health perceptions, this study shows that portals have the potential to allow users to feel more positive about their health. This is an interesting finding because it suggests that information on the patient portal can emotionally engage patients. Researchers and practitioners can strive to understand the nuances in health perceptions because they can lead to higher patient satisfaction scores and potentially higher reimbursement for health care providers.

### Limitations

Despite the many significant relationships discovered in this study, it includes several limitations. We used the AST as a theoretical perspective in this study. Other theoretical perspectives such as the Mere Exposure Effect could be used in future studies to build the research model. Our study included 504 survey responses collected through a cross-sectional survey. Measuring responses through a cross-sectional survey presents concerns of common method bias because the independent and dependent variables are gathered from the same respondent at the same survey session. Although common method bias was not found to be a concern in this study, it is possible that the respondents may not have fully understood the system’s capabilities or may have overestimated or underestimated their use habits. In addition, survey respondents who participate in web-based surveys may be more technologically savvy than the general population. Some of the measures were adapted to the patient portal context, and some new items were created to measure the constructs. Although we followed a rigorous process of survey development and testing, specific follow-up to expand and generalize these definitions is needed. In this study, we adapted the HCAHPS measures used in US hospitals as the measure for patient satisfaction. As the scale used ranges from *never* to *always*, the relationship between the hypothesized constructs and patient satisfaction may be reflective of frequency-based measurement. Future studies can choose to pursue a different scale to measure patient satisfaction. Furthermore, the HCAHPS measures were adapted to suit the context of this study because we sought respondents who had visited their regular health care facility and used an electronic patient portal in the last 12 months. We acknowledge that patient satisfaction can be measured using different methods. Future studies can modify the existing measures or introduce new ways to measure patient satisfaction. We used health anxiety as a proxy for measuring the health status of the patient. Future studies can directly measure the emotional and physical health of the patient to use as control variables, taking into account the response bias with such measurements. Furthermore, this study did not include chronic disease status, health care use, digital literacy, and overall internet use as part of the analysis. Finally, a novel data collection approach that allows actual feedback of users’ habits would greatly illuminate this discussion on patient portals.

### Implications for Research

This study presents several important opportunities for future research. First, the study of gratification has an extensive history [42,65]. This research points to a pivotal role played by gratification in mediating all 3 patient satisfaction outcome variables used in this research (care team interaction, atmosphere, and instruction effectiveness). Future work combining the Uses and Gratification Theory and other constructs such as digital self-efficacy may yield important insights and help to continue the expansion of knowledge in this area.

Second, this research highlights the opportunity to take the validated survey results of self-reported data and determine a way to attempt a similar study using actual reports of user actions from the information systems themselves, rather than reports from patients. This could provide either strong confirmation of this study or yield important new research streams into how the cognitive and affective variables actually affect patient satisfaction and postadoptive use.

Third, efforts to specifically facilitate or enable health self-awareness and health perceptions offer the potential to greatly expand the understanding of patient satisfaction research. Other constructs that might have an interaction effect on postadoptive use and health self-awareness, in particular, offer the potential to shed light on key variables within the nomological network of patient satisfaction.

### Implications for Practice

Our study has several important practical implications regarding the postadoptive use of patient portals, patient perceptions, and patient satisfaction. First, our study findings clearly establish a relationship between patient portal use and patient perceptions. This implies that efforts to increase patient portal enrollment by health care systems are worthwhile, especially because portal use improves the perceptions of satisfaction with the care received. There are several ways in which hospitals are rated on care provided: HCAHPS and Net Promoter scores. Therefore, any insights into improving patient perceptions of the care received become critical.

Second, from a logistics standpoint, this research suggests that patient portal use could reduce medical inefficiencies and wasted time for patients and providers alike. Patients can use the time saved by not having to make phone calls to follow up on tests, schedule appointments, and ask questions. Care providers can handle such requests more efficiently through these portals, thereby reducing errors. With the added benefit of improving patient perceptions of care provided, the mutual benefit would be remarkable.

A third practitioner implication lies in the way in which the outcome variable of patient satisfaction was measured. By measuring three factors of patient satisfaction derived directly from standard HCAHPS scoring, this research was able to identify important antecedents to these outcomes, including key variables such as instruction effectiveness. Therefore, health care organizations can focus on emerging techniques to improve their instruction effectiveness, such as coordinating with web-based apps and providing short postcheckup surveys and audio or video instructions.

Finally, given the current reality of the COVID-19 pandemic, patient portals will continue to remain a top priority for health care organizations. Our study showcases the important role played by patient portals, which enable increased exchange of health-related information without requiring face-to-face support. At this juncture, by improving the portals’ capabilities and by engaging patients to use patient portals, health care organizations can enhance health choices, improving patient satisfaction in the process.

### Conclusions

In this study, we sought to address a key research gap in extant studies by studying the link between patient portal use and the different dimensions of patient satisfaction. We also sought to understand the influence of patient perceptions as mediators of the link between patient portal use and patient satisfaction. Our model shows that patient portal use can influence patient satisfaction through the mediating effects of gratification, health self-awareness, and health perceptions. Future research can seek to take a more nuanced perspective on the mediators highlighted in this study. Patient satisfaction is an important outcome for health care organizations. Therefore, the findings of this study can be used by health care organizations and practitioners to promote effective patient portal use and foster patient perceptions to improve patient satisfaction.

## Appendix

Multimedia Appendix 1 Key articles on patient portal use.

Multimedia Appendix 2 Mediation analysis.

Multimedia Appendix 3 Reflective measures model.

## Abbreviations

AST: Adaptive Structuration Theory
AVE: average variance extracted
HCAHPS: Hospital Consumer Assessment of Healthcare Providers and Systems
TAM: Technology Acceptance Model
UTAUT: Unified Theory of Acceptance and Use of Technology
