# Supplementary material for: Effects of Patient Portal Use on Patient Satisfaction: Survey and Partial Least Squares Analysis
Source: J Med Internet Res. 2021 Aug 27;23(8):e19820. doi: 10.2196/19820 (PMC8433860; doi:10.2196/19820)
Supplement: Multimedia Appendix 1 [file jmir_v23i8e19820_app1.pdf]

Multimedia Appendix 1. Key articles on patient portal use.

Study	Theory and methods	Objective	Constructs	Key contributions
Zhong et al [9]	Retrospective observational study of patients using portals (n=46,544)	To study portal function use and impacts on service use and appointment adherence of primary care patients	Portal adoption (account opened and kept activated); use: messaging, laboratory, appointment, and medication functions; outcomes: rates of primary care office visits	• Adoption of portals does not necessarily translate into use.<br>• Patients with active health issues or other factors adopt portals less but use them more.
Graham et al [3]	Two surveys, patients using portals (n=4296)	To study user satisfaction and influence of portal use on medical appointment attendance	Portal use, health system use, no show rates	• Portal use influenced positive patient experience, decreased health system use and rate of missed appointments
Silver et al [15]	TAM[a] and UTAUT2[b]; survey of patients at university health center (n=1142)	To study the factors that influence portal satisfaction	Portal satisfaction, portal use, health-seeking behaviors, social influence, habit, facilitating conditions, effort expectancy, and performance expectancy	• Positive association between portal satisfaction and health-seeking behaviors and portal use and the antecedent conditions from key constructs (TAM and UTAUT2)
McAlearney et al [10]	Qualitative, interviews of patients using portals during hospitalization (n=120)	To understand patient perceptions of inpatient and outpatient portals	Inpatient and outpatient portals, benefits and risks	• Inpatient portals are beneficial for improving care and communicating information.<br>• Convenience, access to information, and better

				engagement in care are benefits
Hoogenbosch et al [20]	UTAUT[c], cross-sectional survey (n=439)	To study portal use prevalence and influence of characteristics on portal use	Performance expectancy, effort expectancy, social influence, facilitating conditions, behavioral intention to use, and demographics	• One-third of the patients using portal expressed patient satisfaction; UTAUT factors influenced portal use
Dumitrascu et al [22]	Retrospective propensity score matching study (n=7538)	To study if patient portal use during hospitalization improves hospital outcomes	Portal use, 30-day readmissions, inpatient mortality, and 30-day mortality	• Patient portal use did not have significant influence on hospital outcomes (30-day readmission, inpatient mortality, and 30-day mortality)
Aljabri et al [1]	Retrospective review of patients with cancer (n=4594)	To study portal adoption and use, associations with selected safety, use, and satisfaction measures for hospitalized patients with cancer	Demographics: age, sex, marital status, income, portal adoption, portal use, disease severity, and residence	• Half of the patients with cancer adopted portal; demographics (eg, age, gender, marital status, and income) and need (eg, nonlocal residence and disease severity) increased portal adoption
Al-Ramahi and Noteboome [23]	Patient ratings in MyChart (n=500), n-gram analysis	To study users' reviews of mobile patient portal and to identify features associated with	User ratings as proxies for user satisfaction, word-level n-grams for analysis	• Key features for portal use relate to ease of use; others include scheduling appointments and calendar. • *Touch ID* is important as well.

		patient satisfaction		
Winstanley et al [13]	Convenience sample of patients (n=88)	To evaluate inpatient experiences with MyChart Bedside (portal)	Ease of use, communication with nurses and physicians, and understanding medications	• MyChart Bedside helped patients to understand medications and communicate with their provider
Fix et al [11]	Qualitative, convenience sample; 4 patient focus groups, and one-on-one interviews	To examine factors affecting enrollment in a patient portal at patient-centered medical homes	Marketing efforts, administrative barriers, and logistical barriers	• Patient portal adoption is worthwhile; diverse stakeholder perspectives need to be taken into account for portal adoption
Lin et al [6]	Randomized controlled trial (n=606)	To study the impact of a patient portal on patients' satisfaction with access to clinic and care; to study communication characteristics (email and telephone calls) from patients to clinic	Portal use, patient satisfaction, and communication	• Portal's convenience for communicating and messaging valued by portal group; increased satisfaction with communication and overall care
Hassol et al [7]	Patients in Geisinger Health System (n=4282)	To evaluate patients' values and perceptions regarding web-based	User satisfaction, ease of use, communication preferences, completeness of	• Positive attitudes toward EHR and satisfaction with completeness and accuracy of medical information, but there are mild concerns

		communication with their primary care providers while accessing EHR[d]	EHR, and accuracy of the EHR	about privacy and confidentiality
Dendere et al [12]	Review article; meta-analysis of other articles	To review studies on patient portals connected with EMR[e], role in patient engagement, and impact on health care delivery for identifying factors and best practices for portal implementation	Patient portals for EMR, patient engagement, and health care delivery	• Categorized studies in input-process-output framework as predictors of portal use and engagement.<br>• Patient portals improve adherence to medication and discovery of medical errors, improving provider-patient communication
Kruse et al [21]	Review article, CINAHL and PubMed databases (n=27 articles)	To identify characteristics of portals deemed favorable and those deemed needs improvement by patients and providers	Positive and negative perceptions	• Positive: patient-provider communication.<br>• Negative: security concerns and user-friendliness.<br>• Review reveals mixed attitudes of patients with chronic conditions regarding portal use.
Goldzweig et al [17]	Review article, PubMed and Web of Science databases (1990-2013)	To study the effect of patient portals on clinical care	Portal use, efficiency, use, and demographics	• Insufficient evidence for patient portal influence on health outcomes, cost, and so on
Rozenblum et al [24]	Review article, PubMed searched	To explore the impact of HIT[f]	HIT use and patient satisfaction	• HIT is a promising tool, but findings regarding

	from inception to May 2010 (n=56 articles)	on patient satisfaction		association of HIT with patient satisfaction are inconsistent

[a]TAM: Technology Acceptance Model.

[b]UTAUT2: Unified Theory of Acceptance and Use of Technology 2.

[c]UTAUT: Unified Theory of Acceptance and Use of Technology.

[d]EHR: electronic health record.

[e]EMR: electronic medical record.

[f]HIT: health information technology.
