# Supplementary material for: Effects of Patient Portal Use on Patient Satisfaction: Survey and Partial Least Squares Analysis
Source: J Med Internet Res. 2021 Aug 27;23(8):e19820. doi: 10.2196/19820 (PMC8433860; doi:10.2196/19820)
Supplement: Multimedia Appendix 2 [file jmir_v23i8e19820_app2.pdf]

Multimedia Appendix 2. Mediation analysis

We followed Lowry and Gaskin (2014) to conduct mediation analysis using SmartPLS and Sobel test. The results of mediation analysis and Sobel test are provided in Table A. The three dimensions of patient satisfaction are Care Team Interaction (CTI), Atmosphere (ATMOS) and Instruction Effectiveness (IE). IV here refers to Post-adoptive use, MED refers to the corresponding mediator, and DV refers to the corresponding dependent variable. To conduct mediation analysis, we validated the significance of the paths between IV to MED to DV with SmartPLS.

In the table below:

- "Direct No Med" refers to the β of the link from IV to DV without the corresponding mediator included.
- "Direct w/MED" refers to the β of the link between IV and DV, with the corresponding mediator included.
- "IV → MED" refers to the β of the link between IV and mediator.
- "MED → DV" refers to the β of the link between mediator and DV.
- The label "SE" refers to standard error.

We then used a Sobel test calculator (available from www.danielsoper.com) and plugged in the values of IV → Mediator and Mediator → DV, along with the corresponding SE values. As shown by the values of the Sobel test statistic (> 1.96) and 2-tailed probability < 0.05, all the mediators – health self-awareness, gratification and health perceptions – mediate the links between post-adoptive portal use and the three dimensions of patient satisfaction. To establish partial or full mediation, we considered the t-values of the "Direct w/MED" values. T-values greater than 1.96 constitute partial mediation, while t-values less than 1.96 constitute full mediation. In our model, gratification fully mediates the link between portal use and patient satisfaction dimensions. Health self-awareness partially mediates the link between portal use and CTI and ATMOS, while fully mediating the link between portal use and IE. Health perceptions partially mediates the link between partial use and CTI, while fully mediating the link of portal use to ATMOS and IE.

Table A

Mediation Analysis and Sobel Test Results

Relationship	Mediation test for Health Self-Awareness (HSA)			Mediation test for Gratification (GRAT)			Mediation test for Health Perceptions (HP)		
	CTI	ATMOS	IE	CTI	ATMOS	IE	CTI	ATMOS	IE
Direct No MED	-0.172	-0.098	-0.046	-0.038	0.038	0.088	-0.144	-0.07	-0.014
Direct w/ MED	-0.154	-0.082	-0.03	-0.154	-0.082	-0.03	-0.154	-0.082	-0.03
IV → MED	0.162	0.162	0.162	0.394	0.394	0.394	0.158	0.158	0.158
MED → DV	0.296	0.265	0.241	0.27	0.288	0.279	0.131	0.19	0.227
IV → MED SE	0.041	0.041	0.041	0.037	0.037	0.037	0.037	0.037	0.037
Med → DV SE	0.064	0.06	0.057	0.056	0.046	0.055	0.05	0.058	0.054
Sobel Test Stat	3.00417918	2.94478923	2.88684932	4.39219366	5.39713124	4.5796409	2.23317719	2.59916054	2.99572493
1-tailed prob.	0.00133149	0.00161587	0.0019456	0.00000561	0.00000003	0.00000233	0.01276863	0.0046726	0.00136897
2-tailed prob.	0.00266298	0.00323175	0.00389121	0.00001122	0.00000007	0.00000466	0.02553726	0.00934521	0.00273793
Mediation	Partial	Partial	Full	Full	Full	Full	Partial	Full	Full

Note: IV=independent variable, DV=dependent variable, MED=mediating variable, PAU=post adoptive use, HSA=health self-awareness, GRAT=gratification, HP=health perceptions, CTI=care team interaction, ATMOS=atmosphere, IE=instruction effectiveness.
