# Supplementary material for: Effects of Patient Portal Use on Patient Satisfaction: Survey and Partial Least Squares Analysis
Source: J Med Internet Res. 2021 Aug 27;23(8):e19820. doi: 10.2196/19820 (PMC8433860; doi:10.2196/19820)
Supplement: Multimedia Appendix 3 [file jmir_v23i8e19820_app3.pdf]

Multimedia Appendix 3. Reflective measures model.

We tested the structural model with a reflective measures model for patient satisfaction [15, 20, 28, 29]. Although we diverged from reflective measure conceptualization in this study, we collected data on reflective measure in addition to the HCAHPS measures in our survey. The reflective measure used for patient satisfaction in this model is: *I am satisfied with my health care facility.*

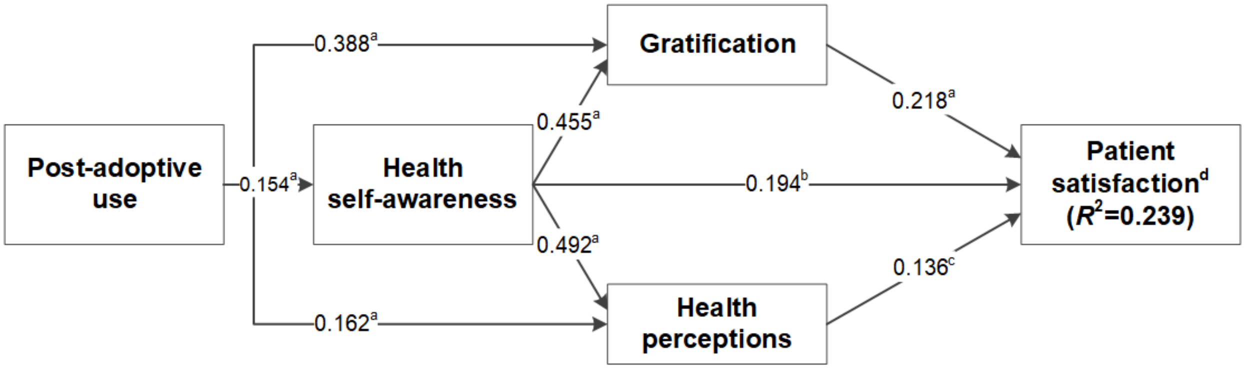

Figure 2.1. Structural model of influences of post adoptive use (Reflective measures Model)

[a]P<.001

[b]P=.006

[c]P=.005

[d]Patient Satisfaction measured using reflective measure

As shown in Figure 2.1, Post-Adoptive use had a positive influence on Health self-awareness, Gratification and Health Perceptions (Measurement model shown in Table 2.1). Health self-awareness positively influenced Gratification and Health Perceptions. Most importantly, Health Self-Awareness, Gratification and Health Perceptions have a positive influence on Patient Satisfaction. Therefore, the findings from the reflective measures model are consistent with the second-order model, highlighting the mediating role played by Health Self-Awareness, Gratification and Health perceptions.

Table 2.1

Measurement model validation

Constructs	Mean	S.D.	PSAT	GRAT	HSA	HP	PAU
PSAT	5.95	1.25	**1.00**				
GRAT	5.32	1.41	0.363	**0.930**			
HSA	5.89	1.16	0.407	0.515	**0.925**		
HP	5.19	1.31	0.316	0.384	0.517	**0.868**	
PAU	3.54	1.47	0.084	0.458	0.154	0.238	**0.854**

Notes: PSAT – Patient Satisfaction measured using one item: I am satisfied with my health care facility, GRAT – Gratification, HSA – Health Self-Awareness, HP – Health Perceptions, PAU - Post-Adoptive Use; Diagonals provide square root of AVE. SQRT of AVE for PSAT is 1 as PSAT is measured using one item. Off-diagonal elements show correlation between constructs.
